# Uncovering commercial activity in informal cities

**DOI:** 10.1098/rsos.211841

**Published:** 2022-11-02

**Authors:** Daniel Straulino, Juan C. Saldarriaga, Jairo A. Gómez, Juan C. Duque, Neave O’Clery

**Affiliations:** ^1^ Centre for Advanced Spatial Analysis, University College London, London, UK; ^2^ Mathematical Institute, University of Oxford, Oxford, UK; ^3^ Research in Spatial Economics (RiSE-Group), School of Applied Sciences and Engineering, Universidad EAFIT, Medellín, Colombia; ^4^ i2t Research Group, Department of Communication and Information Technologies, Universidad Icesi, Cali, Colombia

**Keywords:** labour informality, data for development, computer vision

## Abstract

Knowledge of the spatial organization of economic activity within a city is a key to policy concerns. However, in developing cities with high levels of informality, this information is often unavailable. Recent progress in machine learning together with the availability of street imagery offers an affordable and easily automated solution. Here, we propose an algorithm that can detect what we call *visible establishments* using street view imagery. By using Medellín, Colombia as a case study, we illustrate how this approach can be used to uncover previously unseen economic activity. By applying spatial analysis to our dataset, we detect a polycentric structure with five distinct clusters located in both the established centre and peripheral areas. Comparing the density of visible establishments with that of registered firms, we infer that informal activity concentrates in poor but densely populated areas. Our findings highlight the large gap between what is captured in official data and the reality on the ground.

## 1. Introduction

The world is becoming more urban every day. According to the United Nations, 55% of the world’s population is concentrated in urban areas, a figure that is likely to increase to 68% in the next 30 years [1]. This process presents enormous social, economic and environmental challenges, particularly in the Global South, where most of the growth in urban population will take place. Despite high costs arising from congestion and density, it is widely accepted that the success of cities arises from dense social networks and access to a diversity of opportunity [2,3]. Most obviously this relates to access to jobs.

The spatial organization of jobs and economic activity within developing cities is a core topic of concern across a large number of policy areas. Most straightforwardly, hubs of economic activity require services and transport connections to thrive [4]. These connections facilitate, among other things, better labour market matching between workers and firms and increase the accessibility of services and products to a wider catchment area [5]. Knowledge about job and firm density is also critically important in other domains such as urban planning and disaster resilience. However, in many developing city contexts, dominated by enormous informal sectors, data on the number, type or location of jobs and opportunities are either scarce or incomplete.

According to the International Labour Organization, more than 60% of the world’s working-age population works informally. Most informal workers are found in developing countries where the proportion of informal workers is even higher, in some cases surpassing 90% [6]. Despite the size of the informal sector, the difficulty in tracking and reaching informal firms and workers means that there exists relatively little data on informal businesses outside of survey data (e.g. [7]) such as economic censuses. Examples include a firm census produced for Cali, Colombia, which includes both formal and informal manufacturing firms [8]. However, these are typically very expensive to collect and tend to be incomplete and suffer from reporting biases [9].

In response to these critical gaps, a burgeoning literature has emerged looking to infer—both formal and informal—economic data from alternative sources. A large number of recent studies rely on imagery data which have recently become widely available, e.g. Landsat imagery, Google Maps, Kartaview. A well-known example is satellite night-time light images, which have been used to estimate income growth [10], productivity [11] and urban growth [12]. Satellite data have also been used to predict consumption expenditure and wealth [13], estimate enterprise counts [14], monitor the growth of informal settlements [15,16] and intra-urban poverty [17] (see [18] for a review).

Geo-tagged imagery, such as Google Street View, allows for a more granular picture of the urban environment than satellite imagery [19], providing details on the built environment, street commerce, traffic, etc. Nevertheless, extracting this information is not straightforward. The earliest efforts to deploy it relied on crowdsourcing to e.g. identify the patterns of recovery after Katrina [20], conduct neighbourhood audits [21] and map perceptions of safety and diversity [22]. More recently, advances in computer vision have been exploited to efficiently analyse large numbers of images to monitor pedestrian numbers [23], estimate the demographic make-up of neighbourhoods [24] and classify buildings according to use [25,26] or typology [26,27].

Here, we propose a methodology to automatically detect and geo-reference the presence of commercial activities in a city. By using deep learning, we show that it is possible to efficiently identify what we call *visible establishments* from Google street imagery. These are establishments that are easily identifiable as such at street level and include personal services, retail and amenities (bars, restaurants, etc.) and are sometimes referred to as ‘street commerce’ [28]. We detect both formal and informal commercial firms (our detector does not distinguish between the two). In contrast to some other parts of the world, informal activity is widely accepted and tolerated in Colombia, and hence, small informal firms, even without signage, are typically easily visible from the street. What we detect is a subset of informal activity, however, as a large share is conducted by self-employed persons or in homes and is outside the scope of this study.

Specifically, we use a manually labelled dataset of over 2000 panoramic images sourced from the metropolitan area of Medellín, Colombia, to train a neural network. We then use this algorithm to produce a dataset including the locations of over 170 000 visible establishments across the city. It is important to note that this methodology does not rely on detecting signs, which might not be present for certain businesses, but does rely on the overall appearance of the facade, which includes exposed merchandise, architectural features and other signifiers of commercial activity. Furthermore, it does not distinguish between formal and informal firms, capturing both at once.

We illustrate how our methodology can be applied to investigate a number of questions including identifying economic clusters and the interplay between formal and informal commercial firm clustering, the socio-economic and industrial profile of areas dominated by informal commercial firms and the adherence of informal commercial firms to land zoning rules. While we do not conduct in-depth studies of these issues here, we aim to illustrate how the methodology could be deployed in these domains and prompt future research.

### 1.1. Medellín, Colombia

We focus on the metropolitan area of Medellín (hereafter Medellín) as defined by the National Administrative Department of Statistics (DANE) in their most recent census (see electronic supplementary material, appendix A), spanning 10 municipalities and home to 3.5 million people. Medellín provides a valuable case study for our approach. Characterized by complex industry and international tourism alongside high levels of social segregation and informality [29,30], it is a city of contrasts. In the last 25 years, it has been subject to a dramatic urban transformation, particularly in the form of an innovative public transport system that includes cable cars to reach mountainous communities, which has attracted the interest of the international community [31]. Nevertheless, a majority of Medellín’s economy can still be classed as informal. Estimates put the number of unregistered firms in Colombia above 50% [32], and the share of the working-age population employed in formal employment standing at 44% for Medellín [30] (2015 data).

While there are official registries of firms in Medellín, the large proportion of informal economic activity suggests that a large fraction of commercial activity is not captured by the official data. Our methodology allows us to extract the location of visible establishments across the diverse (both economically and geographically) landscape of Medellín and compare the resulting spatial distribution of ‘street commerce’ with the distribution of commercial firms in the official registry. In this way, we are able to explore, on the one hand, the limitations of official data and, on the other hand, infer the areas where informal (non-registered) establishments concentrate.

Firms and establishments do not have a one-to-one correspondence (as some firms might run more than one establishment), but very few formal firms are multi-establishment firms with branches or plants dispersed over the metropolitan area. While we cannot provide a precise estimate due to a lack of detailed data linking firms to establishments for Medellín (or indeed other Colombian cities), we can draw fairly accurate inferences from the broader literature and some estimates for Colombia and Medellín. Considering all types of firms, studies report that the percentage of firms with multiple establishments ranges from 4% in the USA [33] to 8% in Germany [34]. When it comes to retail, data from Korea [35] suggest that 9% of firms are multi-establishment, while for manufacturing firms, studies report that the percentage ranges from 12% in Canada [36], 8% in Mexico [37], 5% in Indonesia [38] and 3% in Colombia [39] to virtually none in Ghana [40]. By using firm-level data from Colombia’s social security data (PILA) in 2015, which provides establishment locations at a municipality level, we find that just 4% of firms have establishments in more than one municipality, increasing to 7% for commercial activities. These percentages hold when constraining the data to just Medellín, which has 10 municipalities. By focusing on the density of registered commercial firms and visible establishments instead of on simple counts, we are able to draw a meaningful comparison, but it is nevertheless an important caveat for our analysis.

### 1.2. Firm clustering

While data on the spatial location of establishments can be deployed for a very wide range of uses, we illustrate here how it can be used to uncover the patterns of concentration of economic activities in cities. This topic has been studied since the 1820s when cities were thought to consist of a dense central business district (CBD) surrounded by rings of progressively cheaper land [41,42]. More recently, this monocentric view has given way to a polycentric model [43,44] consisting of multiple urban economic cores arising from increased mobility, suburbanization and the movement of manufacturing to the periphery. Polycentrism is particularly acute in cities with weak internal transportation links [45]. A number of studies suggest that global cities have become more polycentric over time [46], while evidence from the USA suggests that polycentric metropolitan areas are larger and more dense [47].

The forces behind firm clustering and agglomeration in cities have been long studied. Benefits include easy access to customers and suppliers, shared labour supply and benefits from knowledge spillovers [2,48,49]. Costs include the cost of land and wages, which are likely to be higher in dense cities [2]. Distinct agglomeration patterns have been observed for manufacturing and services industries, with the latter benefiting from lower land costs and greater returns on access to labour and knowledge spillovers [50,51]. Less is known about agglomeration forces, however, at a finer within-city spatial scale [5]. A small but growing number of studies suggest that agglomerative forces such as knowledge spillovers and inter-firm learning—key to the success of service firms—decline heavily with distance within cities (as reviewed in [52]), thus providing a clear rationale for further research on the topic [53].

A related literature focuses on the role of amenities in attracting people to cities [28,54]. The ‘consumer city’ view sees the presence of amenities as drivers of growth and wages alongside traditional agglomeration economies [55]. In general, the presence of retail amenities is strongly dependent on the size of local population [56], while the share of specialized amenities correlates with the city size [55]. Central place theory provides a framework to understand the spatial organization of amenities in cities from a consumer perspective [57]. The theory posits that amenities are organized in a manner in which multiple ‘urban centres’ serve consumers in the surrounding catchment area. A hierarchy of centres emerges as higher value goods and services, which are needed less frequently and for which customers will travel, concentrate in fewer clusters with large catchment areas. A large body of subsequent literature has adapted this approach, often adopting a less hierarchical polycentric-type model, e.g. [58]. Evidence from China suggests that polycentric cities enjoy more numerous and diverse amenities [59].

Clustering and spatial agglomeration patterns have been less studied in developing city contexts. In particular, little is known about how agglomeration forces shape the locational decisions of informal firms [60,61]. While the prevalence of polycentric cities is a well-established feature of developed cities, some studies have suggested that this model is also well suited to Latin American cities for which formal employment is often distributed between a dense Central Business District and a few, relatively close, additional employment clusters (see [62] for a review). Equipped with our new geo-referenced dataset of visible establishments, we can investigate whether the commercial activity of Medellín follows these patterns.

We note that, while traditionally economic clusters have been detected using geo-located employment data, an absence of such data in many developing contexts has recently prompted researchers to infer the presence of economic clusters from a variety of alternative sources. For example, Lall *et al*. [63] deploy data on building height to infer the internal economic structure of a large set of global cities, while a range of studies deploy population data [64], night-light imagery [65] or point of interest (POI) data (such as Open Street Map or Google Maps) [66] to detect economic clusters. In our case, we rely on the presence of visible commercial firms to detect economic clusters, without any information on employment density, which is more likely to detect clusters with many smaller or informal firms.

Specifically, using spatial auto-correlation analysis, we identify clusters of establishments as well as the relative strength of these clusters. We repeat this analysis for the official firm registry; in this way, we can probe the validity of the polycentric model for both visible establishments and registered firms in Medellín. Our analysis challenges previous work based on land values suggesting a monocentric structure in Medellín [67].

### 1.3. Formal and informal firm interactions

There is a limited existing literature on the interplay between formal and informal commerce. While it was originally thought that the informal economy operated independently from the formal [68], the current consensus is that there are strong links between them [69]. A number of studies find benefits for informal firms (productivity) and workers (wages) from urban density [70]. Focusing on studies for Colombian cities, Duranton [71] and Garcia [72] deployed household survey data to show a positive effect of agglomeration (population and employment density respectively) on wages of informal workers. More related to our work, using data on manufacturing firms in Cali, Colombia, Moreno-Monroy & García [8] deployed spatial analysis to show that formal and informal manufacturing enterprises of similar sizes and industries tend to co-locate, but not necessarily in the same location.

A small number of recent studies have shed some light on the nature of linkages between formal and informal firms, thought to hold the key to agglomeration economies in this context [73]. These studies highlight the role of buyer–seller linkages and firm networks [74,75] as well as the potential for technological/knowledge spillovers leading to learning and innovation for informal firms, as reviewed in [74]. Most relevant for commercial activities of the type we study, formal and informal firms often share a customer base [76,77] and are thought to compete when of a similar size and characteristics [78]. Other potential drivers of agglomeration in the informal sector in developing countries, including Colombia, are commuting costs and property prices [72,79]. These studies suggest that there is a non-trivial relationship between the formal and the informal economy, with the informal sector often benefiting from the presence of formal firms (and probably vice versa).

By considering the difference between the distribution of visible establishments and registered firms, we are able to infer areas where informal establishments concentrate. In other words, a region with a high density of visible establishments which is not reflected in the concentration of registered firms is likely to have a large number of unregistered (or informal) establishments. We show that in Medellín, registered firms exhibit a significantly higher level of clustering than visible establishments and are largely absent from much of the broader metropolitan area. When we focus on the areas with large concentration of informal establishments, we observe two patterns. On the one hand, we find that informal establishments cluster around the formal clusters. On the other hand, we also find them in the areas of the city where there is little presence of registered firms. The first finding aligns with the literature that argues that informal firms benefit from proximity to formal firms. The second finding suggests that they also perform a substitute role in more remote areas, which might not be as attractive for formal firms.

### 1.4. Neighbourhood characteristics

A large literature focuses on social, cultural and economic segregation in cities (e.g. [80,81]). There is convincing evidence that lower income groups tend to make more purchases at informal firms [82] and that rising incomes lead to a lower propensity to consume informal sector goods [77]. To study the distribution of informal establishments with respect to poverty, we turn to a classification of neighbourhood blocks into strata by the Colombian government. This classification, sometimes controversial, aims to progressively price services such as water and rubbish collection and has been previously used to proxy for poverty in a variety of studies (e.g. [29]). By using this classification, we observe a significant correlation between local socio-economic status and the presence of unregistered establishments; poorer areas (and those with higher population density) have a higher density of informal commerce.

While, at this stage, we cannot assign a type of commerce to each firm, we can investigate the industrial profile of the formal sector in each neighbourhood. While the nature of informal firms remains a matter of debate, with some authors focusing on the role of managerial talent [83], a new perspective sees informal firms as low-complexity operations requiring few specialized skills, while formal firms require larger teams of specialized workers [30]. We find that informal establishments concentrate in areas with low-complexity formal firms, thus limiting learning and knowledge exchange opportunities with more sophisticated formal firms.

### 1.5. Land use

Locational information on visible establishments allows us to explore dynamics beyond the concentration and distribution of commercial activity. One of the main tools in the city planner’s arsenal is the creation and implementation of land use plans [84,85], which are intended to guide the growth and development of the city. While the effectiveness and bias of these plans is a hotly debated topic [86,87], there appears to be a consensus that non-adherence to zoning restrictions is more prevalent in poorer neighbourhoods [88]. By comparing the location of visible establishments to the land use set out by Medellín, we are able to challenge this perception, finding a more nuanced picture whereby non-adherence to zoning rules is widespread across socio-economic strata, and that while more common in our set of visible establishments, it is also high for registered firms.

The remainder of this article will take the following format. First, we introduce our methodology for detecting visible establishments in geo-referenced street view images. We then use this dataset to explore the dynamics of clustering and agglomeration of street commerce in Medellín, as well as to investigate which areas appear to have a larger presence of informal businesses. We then use the same dataset to map the non-adherence of establishments to land use regulations. Finally, we discuss the implications of our work as a new way to gather establishment-level data on commercial activity, as well as its limitations.

## 2. Results

### 2.1. A new method to uncover visible commercial activity in data scarce contexts

Here, we propose an algorithm based on machine learning applied to Google Street View images that enable us to identify the location of what we describe as visible establishments, i.e. commercial establishments that are easily identifiable from the street. For any region for which such images are available, this approach will produce a geo-referenced database of visible commercial establishments. Here, we apply the algorithm to the metropolitan area of Medellín, Colombia. A high level of informal economic activity and socio-economic diversity, as well as a rugged topography and a non-homogeneous urban sprawl, combine to present a challenging case study for our detector. While our analysis focuses on Medellín, the methodology is straightforwardly transferable to other cities and regions.

**Figure 1.**
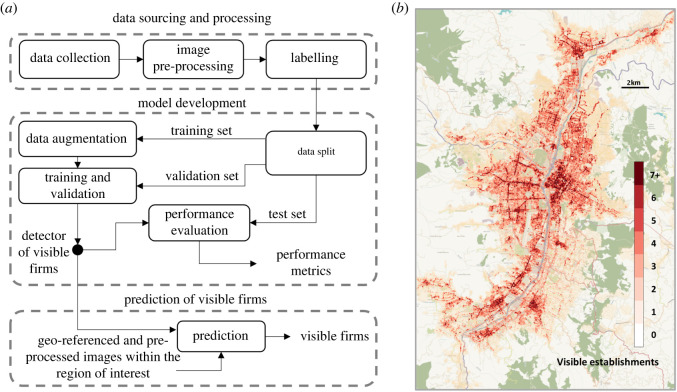
Detecting visible establishments. (*a*) The workflow for detecting visible establishments has three main stages. During data collection, the images necessary for training are acquired, processed and labelled. In the model development stage, the model is trained on an augmented dataset and the best hyper-parameters are found using a validation set, while the performance metrics are computed using a an (unseen) test set. Once the detector has been fitted, we apply it to the region of interest, in this case Medellín. (*b*) The set of visible establishments in Medellín. The detection algorithm found over 170 000 visible establishments in the metropolitan area; while they concentrate around the city centre and along some of the busiest streets, they are present across the whole metropolitan area.

The workflow employed to build the algorithm is illustrated in figure 1*a*. To create a training set for our detection algorithm, we randomly sampled 2000 points from the street network of Medellín (illustrated in the electronic supplementary material, appendix S1), which we sourced from Open Street Maps (OSM), an open source project that freely provides road networks and other useful geographical information. By calling the Google street view API, we obtained a panoramic image for each of these points, which we then transformed into two standard images, one for each side of the road. These images were manually labelled by drawing a bounding box around every facade and then labelling each facade as either commercial or non-commercial. The labelled dataset contains approximately 2000 commercial facades and approximately 6000 non-commercial. The images were randomly split into training (60%), validation (20%) and test (20%) sets. The training set was then enriched following standard data augmentation procedures such as rotation, translation and noise addition [89], which are common in detection and classification tasks.

Three architectures were considered for the detector: single shot detection (SSD) [90], you only look once (YOLO) [91] and Faster R-CNN [92]. These detectors take an image as the input and output the location(s) of the object(s) of interest within the image, which in our case are the facades of commercial firms. Given that we only had a small training set, we used transfer learning [93], which allowed us to retrain models that had been previously fitted for a similar object detection task. The three different architectures were trained using our training and validation sets, and their performance was compared using the test set.

As well as training the detector, it is necessary to identify a set of locations from which panoramic images will be sampled to fully cover the urban area of Medellín. To do so, we obtained the street network of Medellín from OSM and chose points in such a way that the images would not overlap while also capturing (almost) all the facades. Since after processing a 360 panoramic image we obtain roughly 20 m of each side of the street, we wanted to ensure that along each road segment we had an image every 20 m. To do so, we first took all of the network crossings. For each segment of the network between two crossings, we added the maximum number of points we could while ensuring they remained at least 20 m apart. This heuristic allowed us to quickly create a set of points that gives us close to maximal coverage of the city. We then used Google’s street view API to obtain a panoramic image for each point. Finally, we applied the detector to each image to produce a list of geo-referenced points with counts of detected visible firms.

The performance of the algorithm is summarized in table 1. The precision score (i.e. the proportion of commercial facades that are correctly identified) is very high (greater than 97%) in both the validation and the test subsets, indicating that almost all the detections are visible establishments. The recall (the proportion of visible establishments that are detected) is lower, around 60%, and thus, some visible establishments are likely to go undetected. This performance is comparable to related work [94,95], which obtained scores of around (85%, 65%) for precision and recall. We also show the F1 score which is the harmonic mean of the precision and recall scores.

**Table 1.** Performance of the algorithm.

score	training	validation	test
precision	0.9965	0.9706	0.9816
recall	0.8871	0.6094	0.5941
F1	0.9386	0.7487	0.7402

To obtain a consistent picture of the number of visible establishments, we exclusively used street view images captured in 2017. Figure 1*b* shows the full set of 170 000 visible establishments found by the detector superimposed on the metropolitan area of Medellín, shown in orange. As expected, concentrations of firms are clearly visible in this dataset. But we also notice that the footprint of the detections extends across most of the urban area.

While the algorithm will miss a fraction of the true set of all visible establishments (see recall score mentioned earlier), the overall spatial distribution is robust with respect to random omissions (see electronic supplementary material, appendix B).

### 2.2. Visible establishments cluster around five centres in Medellín

To investigate spatial clustering in the distribution of visible establishments, we first estimate the density of visible establishments across the metropolitan area. The density effectively smooths errors in the data, which mainly arise from distances between the point an image was taken and the precise location of a firm. By using a grid, we divide the region into cells of 200 m by 200 m (about the size of a city block), which determines the granularity of the density we will obtain. By applying kernel density estimation [96] to the set of visible establishments, we obtain *ρ*_vis_, which is shown as a red surface in figure 3*b*. This density can be interpreted as a spatial probability distribution for a firm sampled at random from our detections.

**Figure 2.**
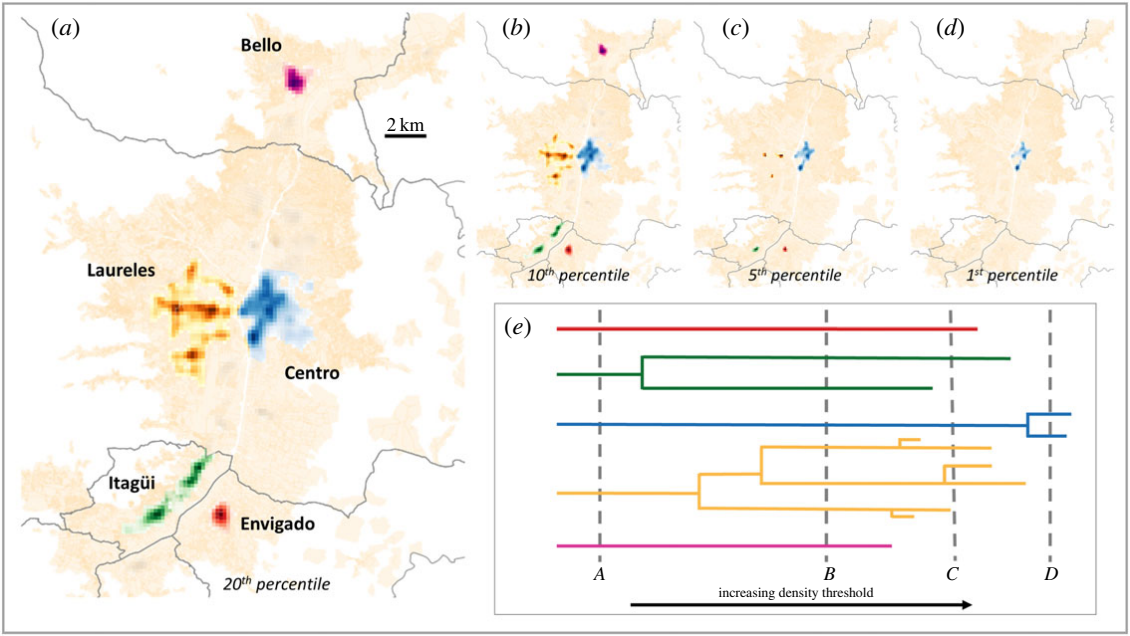
Clustering of visible establishments. We apply local indicators of spatial association (LISA) statistics to identify clusters of visible establishments across the metropolitan region. (*a*) When we include the top 20% of cells by density value, there are five distinct clusters (city centre (Centro), Itagüi, Bello, Laureles, and Envigado). (*b*–*d*) By changing the density threshold at which we include cells in the analysis, we can distinguish clusters by the ‘strength’ of clustering and identify sub divisions of clusters into smaller, more concentrated, agglomerations. (*e*) A dendrogram illustrates the emergence and merging of clusters as the density threshold changes.

Equipped with the density, we applied local indicators of spatial association (LISA) [97] statistics to identify clusters of visible establishments across the metropolitan region (similar to [47]). For each cell, LISA performs a statistical test of the spatial auto-correlation of *ρ*_vis_. The resulting *p*-values can be used to decide which cells show a significant clustering pattern. A significance level of *p* = 0.1 is commonly used as a threshold for identifying employment clusters [47]. In figure 2 we adopt this *p*-value (with sensitivity test in electronic supplementary material, appendix D), but vary the density value at which cells are included in the analysis. In other words, we vary the minimum density value (*ρ*_vis_), which is required for grid cells to be included in the LISA analysis. By increasing this threshold, we only include cells with a higher firm density and hence we would expect to detect fewer, smaller, but denser, clusters.

Figure 2*a* shows that when we include the top 20% of cells (or higher, see electronic supplementary material, appendix D), there are five distinct clusters (city centre (Centro), Itagüi, Bello, Laureles and Envigado). Both the city centre and Laureles are located in the municipality of Medellín, while the other clusters are located in the old town centres of their respective municipalities. These clusters constitute evidence of the polycentric nature of Medellín in which several urban cores operate as economic engines within the city.

We vary the density threshold to further investigate the spatial concentration of visible establishments, in particular to distinguish clusters by the ‘strength’ of clustering and to identify subdivisions of clusters into smaller, more concentrated, agglomerations. We can illustrate the evolving structure of clusters for different density thresholds via a dendrogram shown in figure 2*e*. The visualization reveals at what threshold value a cluster appears or merges with other clusters, thus uncovering substructures within clusters.

We observe that as the threshold increases, Bello (a poor suburb in the north of Medellín) has the weakest clustering and is the first to disappear. In contrast, the Centro cluster is the strongest and persists as the threshold increases. At very high values of the density threshold, only Centro remains. Laureles exhibits substructure as it fragments into several clusters (*b*,*c*) before disappearing (*d*). Similarly Itagüi splits into North and South, which remain present until Itagüi North disappears (*c*) followed by Itagüi South and Envigado (*d*).

There is no doubt that the nature of the establishments we capture tend to be service oriented and that these tend to locate close to customers, particularly when cities are less well connected. Although recent investments in public transport have yielded a well-connected metropolitan area, with travel times from Bello to Envigado cut from over 2 h to just 30 min since the mid-1990s, it appears that economic activity remains highly distributed. This is probably in major part due to a path dependence in the development of economic clusters. In particular, notice that even though the area covered by the clusters increases as we increase the threshold, the clusters never cross municipality lines (shown in grey). Apart from Laureles, the clusters are located in the old centre of the municipalities. Hence, it appears that path dependence in the expansion of commercial activities has resulted in a modern configuration of economic clusters along historic municipal lines.

The previous work on identifying commercial clusters in Medellín [67] used the land value of the locations of registered firms, aggregated to neighbourhood level, to produce clusters of industrial activity and services. The authors identified a single commercial cluster within the municipality of Medellín (they did not consider the wider metropolitan area), which contrasts with our analysis that uncovers two distinct clusters in this area, Laureles and Centro, which are separated by the river. Hence, our approach, which does not depend on official data and is not restricted by a particular geographical unit, produces more finely grained results.

### 2.3. Commercial informal (visible but non-registered) establishments lie in the shadow of registered firms

While the previous work [67] has investigated the presence of economic clusters for the municipality of Medellín, this study omits the broader metropolitan area. Here, we probe to what extent existing data on the location of formal registered firms captures the economic geography of Medellín. To do this, we exploit the Colombian Statistical Directory of Companies (DEE, Directorio Estadístico de Empresas), a dataset that contains the location of all firms registered with DIAN (Dirección de Impuestos y Aduanas Nacionales, the Colombian tax authority).

This dataset contains not only the location of each firm but also its industry code (at four-digit level). The number of registered firms in the metropolitan area is around 150 000, which is smaller than the number of visible establishments (170 000). Note, however, that this dataset covers firms, not establishments. Furthermore, the space of registered firms and visible establishments datasets overlap in the sense that the visible establishments dataset contains both formal (registered) establishments and informal establishments. Hence, a subset of establishments—those that are both formal/registered and commercial—should appear in both datasets. Alongside the full set of registered firms, we use a list of industries labelled as ‘street commerce’ in [28] to create a subset of registered firms (approximately 15 000 firms) termed ‘registered commercial firms’. These are active in industries that are likely to be visible from the street (see electronic supplementary material, appendix C for the list of industries). We note that this subset is an order of magnitude smaller than the visible establishments dataset, further signalling the extent of missing commercial activity in official data.

**Figure 3.**
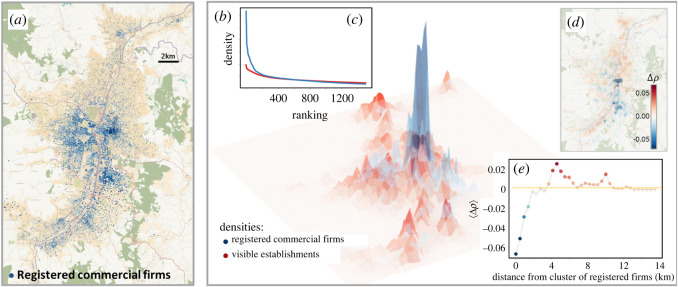
Visible and registered firms. (*a*) The location of registered firms in Medellín, where each firm is represented by a single blue dot. (*b*) The surface represents the spatial density of visible (*ρ*_*vis*_ in red) and registered commercial firms (*ρ*_reg_ in blue). The peaks of the blue distribution are much more pronounced, but the red distribution extends across the metropolitan area. (*c*) Ranking the cells according to density allows us to see how much more spatially concentrated registered firms are; the cells corresponding to the top percentile (1%) accounts for over 20% of the registered firms but only 13% of the visible establishments. (*d*) We calculate Δ*ρ* := *ρ*_vis_ − *ρ*_reg_, the surplus density of visible establishments over registered commercial firms. Positive values of Δ*ρ* suggest regions with significant informal commercial activity. (*e*) Informal commercial activity peaks at a distance of 4km from the centroids of the formal clusters (El Poblado and the city centre).

In figure 3*a*, we show the distribution of registered commercial firms in Medellín. We observe that their spatial distribution is different from that of the visible establishments, most notably in the absence of registered firms in the northern poorer communities of Bello and Copacabana. We show the density of both the registered commercial firms *ρ*_reg_ and visible establishments *ρ*_vis_ (correlation of 0.64) in figure 3*b*. The high concentration of registered firms in the city centre is apparent from the large peak in the density. A second region of high density just south of the city centre is also apparent; it corresponds to the new business district (El Poblado) where skyscrapers house the headquarters of many of the largest firms. By contrast, the density of visible establishments is flatter across the urban extent. Hence, we capture a large swathe of economic activity outside the main centres that is not present in official data.

To quantify the difference in concentration, we rank the cells according to both *ρ*_vis_ and *ρ*_reg_. Figure 3*c* shows that the highest ranked cells account for a larger proportion of registered commercial firms than they do for visible establishments. For example, the cells representing the top percentile (1%) account for 19.8% of registered firms but only for 13.7% of visible ones. We quantify the disparity in concentration by calculating the coefficient of variation (CV) for both densities. While the density of visible establishments has a CV = 2.22, the density of registered commercial firms scores 3.03.

Next, we apply LISA analysis as mentioned earlier to the set of registered commercial firms. At the *p* = 0.1 significance level, we find just two formal clusters, Centro and El Poblado (see electronic supplementary material, appendix S2). Hence, when using official data on firms for Medellín, it appears that there exist just two centres of commercial activity (one of which overlaps with the visible establishment clusters, Centro). This is in stark contrast to the five distinct centres that are apparent when LISA is applied to the visible establishments dataset. Hence, we observe limited spatial overlap between concentrations of registered commercial firms and visible establishments outside the city centre, which might suggest an absence of widespread linkages between the formal and informal sectors, as suggested in [98].

We further investigate the spatial concentration of informal activity relative to the formal clusters. While we cannot directly disentangle formal and informal in our dataset of visible establishments, we can look for areas in which there is an ‘excess’ concentration of visible establishments relative to registered commercial firms. To do this, we calculate the difference in density between visible and registered commercial firms Δ*ρ* := *ρ*_vis_ − *ρ*_reg_ (figure 3*d*). Positive values (red) indicate that visible establishments are more concentrated than registered commercial firms.

We compute the mean value of Δ*ρ* as a function of the distance to the formal clusters as shown in figure 3*e*. We observe a peak in the concentration of visible establishments relative to registered commercial firms at around 4 km from the centroid of the clusters. Hence, we find that unregistered or informal commercial firms concentrate in areas surrounding formal clusters. In the following section, we will investigate the characteristics of these areas, including socio-economic status, population density and industrial complexity.

Overall, we find that visible establishments are widely distributed across the urban extent and organized around multiple clusters distributed across the centre, north and south of the city. By contrast, registered formal firms are concentrated in just two central clusters, Centro and El Poblado, which exhibit a surplus concentration of visible establishments relative to registered commercial firms in their surrounding areas.

### 2.4. Informal establishments concentrate in poor but densely populated neighbourhoods with few complex industries

The analysis mentioned earlier suggests that visible establishments tend to concentrate in poorer areas away from the traditional economic centre of the city. Here, we are specifically interested in uncovering the density, socio-demographic status and industrial profile of neighbourhoods, which are home to many visible establishments but few registered firms.

**Figure 4.**
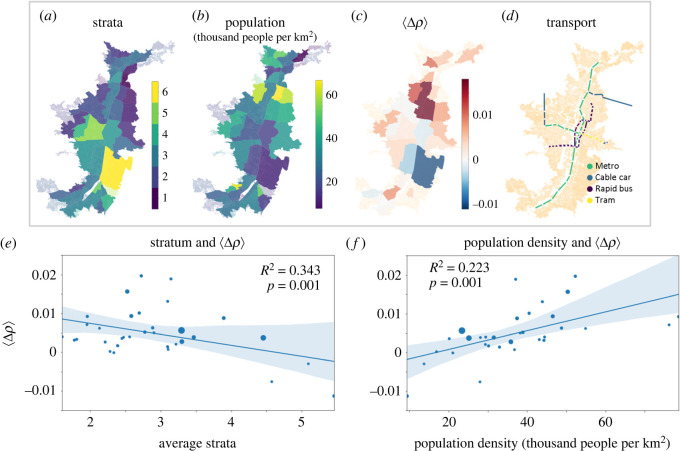
Socio-economic strata and population density. (*a*) The comunas of Medellín according to their strata. Rich neighbourhoods (in yellow) are concentrated in El Poblado, while the outskirts usually belong to the poorest strata. (*b*) The most densely populated comunas are located just north of the city centre. (*c*) We show the average difference in density between visible and registered commercial firms, 〈Δ*ρ*〉, at the comuna level. Those in red have a relatively high density of visible establishments, and those in blue have a larger concentration of registered commercial firms. (*e*) There is a significant negative relationship (*p* < 0.001) between the average stratum of a comuna and 〈Δ*ρ*〉 at the comuna level. (*f*) Similarly, we find a significant relationship between the population density and 〈Δ*ρ*〉 at the comuna level.

Granular data on socio-economic demographics is rarely available for developing cities. Here, we exploit a policy of the Colombian government, which aims to progressively adjust charges for public utilities and services, to infer socio-economic status at a neighbourhood level. As a result of this policy, all neighbourhoods have been classified into six strata with 1 being the poorest and 6 the richest. Medellín has 10 municipalities that are subdivided into 66 comunas. Figure 4*a* shows the mean stratum of each comuna. We observe that the richest stratum concentrates in El Poblado, south of the city centre, while the majority of the city belongs to strata 2–4. While strata do not perfectly correlate with income or other socio-economic variables, it has been widely used as a proxy for socio-economic status in the academic literature [29,99].

We show population density and 〈Δ*ρ*〉 at comuna level in figure 4*b*,*c* and the layout of the metro and bus rapid transport in figure 4*d*. We immediately observe that larger values of Δ*ρ* are associated with lower stratum (poorer areas) but larger population densities. Figure 4*e*,*f* confirms that there is a statistically significant correlation between strata and population density with Δ*ρ* at the comuna level. Hence, poorer comunas with a high population density are home to a higher density of visible establishments than registered commercial firms. Figure 4*d* shows that some of these areas are also well served by the fêted metro system. While all three variables in this analysis—〈Δ*ρ*〉, stratum and population density—show spatial auto-correlation at the neighbourhood level, this auto-correlation is no longer significant when we aggregate to the comuna level (see electronic supplementary material, appendix E where we also show that the residuals of the regressions are not significantly autocorrelated); therefore, it is possible to perform the regressions without adding spatial interactions.

Hence, it appears that informal commercial firms concentrate close to customers in poor but well-connected areas that are not being served by registered firms. This result is consistent with the idea that amenities and commercial firms will tend to locate close to consumers [56,57], and that informal firms have lower barriers to entry that allow them to source workers and meet demand in poorer areas [30,83]. It is also backed up by research on South Africa which cited proximity and convenience as a key driver for customers of informal firms [100].

Next we investigate the presence of visible and registered firms with respect to the local industrial profile. In particular, we would expect to find a higher density of informal firms in neighbourhoods with few sophisticated industries. Conversely, we would expect a higher density of registered firms in neighbourhoods that are home to complex industries such as finance, engineering or law. This is consistent with the argument that informal firms can been seen as those that require small teams with few specialized skills, while formal firms require larger teams with specialized skills found in large dense agglomerations [30].

**Figure 5.**
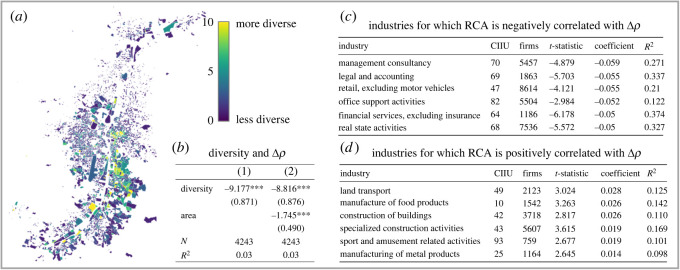
Industrial structure. (*a*) Map showing industrial diversity of neighbourhoods. (*b*) The relationship between industrial diversity and Δ*ρ* at the neighbourhood level. In the second column we control for the size of the neighbourhood. Less industrially diverse neighbourhoods are home to higher concentrations of visible establishments relative to registered commercial firms. (*c*,*d*) For each industry sector (at the two-digit level), we regress Δ*ρ* with (the concentration of the sector) across comunas. We find concentration in complex sectors such as business and legal activities is negatively associated with Δ*ρ* (higher density of registered commercial firms), while concentration in low complexity activities such as manufacturing and construction is positively correlated with Δ*ρ*. RCA, revealed comparative advantage; CIIU, Clasificación Industrial Internacional Uniforme (International Standard Industrial Classification).

Building on an established literature that has shown that complex activities are located in industrially diverse places home to many skills and capabilities [101], we construct a simple proxy for the industrial complexity of each neighbourhood by computing an industry diversity score. It is calculated for each neighbourhood by counting the number of distinct industries (at the four-digit level) that are represented by at least one registered firm. These neighbourhoods are small geographical units consisting of a few blocks. Figure 5*b* shows that there is a statistically significant negative relationship between this score and 〈Δ*ρ*〉 at a neighbourhood level, confirming that less diverse neighbourhoods tend to be home to a higher density of visible establishments relative to registered commercial firms. We show a comparable result at the comuna level in electronic supplementary material, appendix F.

Since diversity is an imperfect metric for the industrial sophistication of a neighbourhood, we investigate the distribution of visible establishments with respect to individual sectors. To do this, for each comuna, we calculate the revealed comparative advantage (RCA) from [102], see Material and methods), a metric capturing industry concentration in a place for each of the 88 industry sectors at the two-digit level. To probe the relationship between the RCA and 〈Δ*ρ*〉 for each sector, we applied weighted regression at the comuna level (weighted by the number of firms in the comuna; we show the unweighted version in electronic supplementary material, appendix G). We show the top and bottom six sectors ranked by the size of the regression coefficient in figure 5*c*,*d*. We find that comunas with a comparative advantage in complex sectors such as business and legal activities are negatively associated with 〈Δ*ρ*〉 (i.e. they have higher density of registered commercial firms than visible establishments), while comunas concentrated in low-complexity activities such as land transport, manufacturing and construction are positively correlated with 〈Δ*ρ*〉. Hence, visible establishments tend to outnumber registered firms in these areas.

### 2.5. Land use zoning is ineffective across all strata

**Figure 6.**
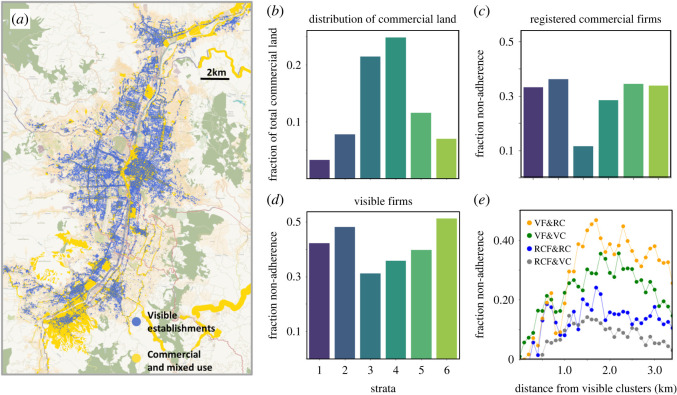
Land use. (*a*) Map of commercial land and visible establishments. (*b*) Distribution of commercial or mixed-use land across strata. (*c*) Non-adherence across socio-economic strata for registered commercial firms. (*d*) Non-adherence across socio-economic strata for visible establishments. (*e*) Non-adherence of visible establishments (VF) and registered commercial firms (RCF) as a function of distance from both visible clusters (VC) and formal clusters (FC). We find the highest level of non-adherence of visible establishments occurs at around 2 km from the centre of the formal clusters, with a lower peak for non-adherence around visible clusters.

Zoning plans have been widely used as a tool for managing urban growth. But it has been pointed out that the enforcement is many times selective [86], that it can reinforce inequality and related dynamics [86,87] and that there is generally a large amount of non-conformance to the plans [103]. Problematically, non-adherence to zoning restrictions is usually associated with poorer neighbourhoods further complicating the debate [88]. Furthermore, work on street commerce and land use suggests that such firms benefit from mixed-use zoning, enabling firms to flexibly locate near consumers rather than designated shopping zones [28]. Here, we investigate adherence to zoning of visible and registered firms by comparing their location with the official land use plan of the city (electronic supplementary material, appendix A) as shown in figure 6*a*.

We investigate the adherence of firms through the lens of socio-economic segregation or stratum. In figure 6*b*, we show that most of the commercial and mixed-use land belongs to the middle strata 3 and 4. We define the non-adherence rate as the fraction of firms in non-mixed or commercial zones as a share of total firms. Consistent with figure 6*b*, we find that non-adherence of registered commercial firms is lower in the middle strata but higher in both poor and wealthy areas (with less available commercial and mixed-use land). Similar patterns are found for the larger set of registered firms, see electronic supplementary material, appendix H. Contrary to common perceptions, however, figure 6*c* shows that non-adherence of visible establishments is reasonably steady across all strata. We find that for all six strata between 30% and 50% of visible establishments are located on non-commercial land. Furthermore, the richest stratum exhibits the highest level of non-adherence, while strata 3 and 4 have the lowest level. Hence, even in wealthy areas, the incentives for commercial firms to locate are stronger than the enforcement of zoning regulations. Overall, adherence to zoning regulations is low for both visible and registered firms across all strata including wealthy areas.

We found from aforementioned discussion that visible establishments tend to concentrate (relative to registered commercial firms) on the outskirts of formal clusters. Here, we investigate the levels of non-adherence of firms relative to their distance to a cluster. Figure 6*d* plots the average non-adherence of visible establishments and registered commercial firms against the distance to the midpoint of the closest formal cluster or visible cluster (here the latter set excludes the overlapping cluster, Centro). We find the highest level of non-adherence of visible establishments occurs at around 2 km from the centre of formal clusters, with a lower peak for non-adherence around visible clusters. Hence, it appears that visible establishments, which include informal commercial firms, are most likely to violate zoning laws when located close to formal centres, probably benefiting from both a combination of a dense customer base and linkages with formal firms. We also consider the non-adherence of registered commercial firms, finding a smaller peak at a similar distance from formal clusters. Finally, consistent with what we would expect, the lowest level of non-adherence with a minimal peak is found for registered commercial firms around visible clusters.

## 3. Discussion

This article proposes a new methodology for identifying and tracking commercial activity in informal cities using street view imagery. This approach is a fast and cost-effective alternative to surveys and commercial registries. By focusing on the metropolitan area of Medellín, we show that the detection algorithm allows us to map the spatial distribution of visible commercial activity and identify economic clusters with a high density of visible establishments. Comparing our dataset with the set of registered firms, we demonstrate that we capture activity that is not reflected in the official records, particularly in poorer and more densely populated regions of the metropolitan area.

Our results contrast with the previous work [67], which combined data on land value with the locations of formal firms to identify just one central commercial cluster. We also find distinct patterns compared with related work [8] that analysed census data on both the size and location of formal and informal manufacturing firms in Cali. While not directly comparable, we detect the presence of visible commercial firms (in the absence of registered commercial firms) across the Medellín metropolitan area, while [8] found that informal manufacturing firms in Cali exhibit higher levels of spatial agglomeration than their formal counterparts, although this does differ by sector.

Our methodology is not without limitations. Firstly, the set of visible establishments is a specific subset of all establishments, as it only includes those that are easily identifiable at the street level. Visible establishments include retail activities, personal services and other similar activities and amenities. These are arguably some of the most dynamic and informal sectors of the economy, and hence, the dataset is thus particularly useful for capturing economic activity in a developing city, and any analysis done on this set of firms must take this into account. Secondly, while comparable to related efforts to detect shopfronts [94,95], the algorithm does not perfectly identify commercial firms. Specifically, while these algorithms show great precision in identifying firms, their recall is not as high, which means they are likely to underestimate the number of firms. Thirdly, although the methodology is easily transferable to other contexts where street imagery is available, it does require training data for the detector. In our case, this involved many hours of manually labelling imagery. Future work will investigate the extent to which new regions require a bespoke training set, or whether images trained on one city can be used to identify facades in another.

There are many other avenues for the future work. Here, we have considered imagery for just 1 year, but analysis of imagery over longer time periods could provide important information about the evolution of the spatial concentration of economic activity over time, and the impact, for example, of public transport and road investments. We cannot easily disentangle formal from informal firms in our dataset, and use a registry of formal firms to identify areas with an ‘excess’ concentration of visible establishments relative to registered firms to infer the presence of informal firms. Future work might aim to further match these datasets, or deploy other techniques—such as training the detector to identify informal firms—in order to further distinguish informal from formal firms. In addition, there are other possible approaches to close the gaps in official data, such as crowdsourced data. Google Street Maps and Open Street Maps, for example, provide information on amenities such as bars, restaurants and shops, and may also provide limited information on the location of a wider set of sectors including some manufacturing establishments. While crowdsourced data are susceptible to self-selection and other biases [104], a potential avenue for future research would be to integrate and benchmark against these other sources.

## 4. Material and methods

### 4.1. Density estimation

Kernel density estimation was applied to the detections in the region of Medellín. A grid of 200 m by 200 m cells was drawn over the extension of the city. The level of smoothing is dictated by the bandwidth which we fixed at 150 m, which is equivalent to walking two blocks (see [105]). For each cell in the grid, we obtain a density value, and we re-scale these values so that they sum up to one.

### 4.2. Clustering

To identify the clusters of visible establishments, we followed the methodology of [47] and applied LISA [97] to our dataset of visible establishments. This method calculates a local version of the traditional Moran’s I auto-correlation statistic. Contiguous cells that show auto-correlation above a certain significance level are grouped into clusters, and a *p*-value of 0.10 is the usual choice in the literature [47]. To investigate the persistence of the clusters, we vary the minimum density required for a cell to be included. The clusters found at each different threshold form a family of nested clusters described by the dendrogram in figure 2.

### 4.3. Socio-economic strata in Medellín

The national census of Colombia [106] provides the official socio-economic stratum at the neighbourhood level. We focus our analysis on the urban region of Medellín, as defined in the census. This region spans 10 municipalities. Municipalities are divided into 66 comunas (or *macrozonas*) which can be further divided into neighbourhoods. The majority of our analysis has been carried out at the comuna level, with the instances in which we use neighbourhoods clearly indicated.

### 4.4. Revealed comparative advantage

The RCA [102], which we calculated at the comuna level, is given by

$$RCA_{i,c}=\frac{\left|F_{c}\cap F_{i}|/|F_{i}\right|}{\left|F_{i}|/|F\right|},$$

where *F*_*c*_ is the set of registered firms in comuna *c*, *F*_*i*_ is the set of registered firms in industry *i* and *F* is the set of all registered firms. If it is bigger than one, it shows that that industry is more concentrated in that comuna than it is in the whole region.

### 4.5. Land use and visible establishments

Land use in Medellín is governed by the Plan de Ordenamiento Territorial (POT) [107]. This plan allocates a fraction of the total land to commercial and mixed use; theoretically, all firms should be located inside these areas. By using the geo-location of the detected firms, we verified which firms were located in this region. We labelled every detection according to the socio-economic stratum in which it falls. For each stratum *i*, we calculated the following measure of (non-)adherence to land use:

$$A(s)=1-\frac{\left|C_{s}\cap C_{A}\right|}{\left|C_{A}\right|},$$

where *C*_*s*_ is the set of visible establishments that belong to stratum *s* and *C*_*A*_ is the set of visible establishments that are located in commercial and mixed-used land.

We repeated this analysis for the set of registered firms and for commercial registered firms, which we obtained from the industries classified as street commerce in [28].

## Data Availability

All data required to reproduce the analysis in the manuscript are available from the Dryad Digital Repository: https://doi.org/10.5061/dryad.0cfxpnw4s [108]. All public datasets used can be found in the references section, and have also been added to the data repository. The data repository also contains all the code required to perform the detections and subsequent analysis presented in the paper. Supplementary material is available online [109].
